# Miniature enOsCas12f1 Enables Targeted Genome Editing in Rice

**DOI:** 10.3390/plants14142100

**Published:** 2025-07-08

**Authors:** Junjie Wang, Qiangbing Xuan, Biaobiao Cheng, Beibei Lv, Weihong Liang

**Affiliations:** College of Life Science, Henan Normal University, Xinxiang 453007, China; 2019093@htu.edu.cn (J.W.); xuanqiangbing@163.com (Q.X.); cbb521970@outlook.com (B.C.); lvbeibei1376@163.com (B.L.)

**Keywords:** genome editing, enOsCas12f1, bases deletion, transcriptional activation

## Abstract

The type V CRISPR/Cas12f system, with its broad PAM recognition range, small size, and ease of delivery, has significantly contributed to the gene editing toolbox. In this study, enOsCas12f1 activity was detected during transient expression in rice protoplasts. The results showed that enOsCas12f1 exhibited DNA cleavage activity when it recognized TTN PAMs. Subsequently, we examined the gene editing efficiency of enOsCas12f1 in stably transformed rice plants, and the results showed that enOsCas12f1 could identify the TTT and TTC PAM sequences of the *OsPDS* gene, resulting in gene mutations and an albino phenotype. The editing efficiencies of TTT and TTC PAMs were 6.21% and 44.21%, respectively. Furthermore, all mutations were base deletions, ranging in size from 7 to 29 base pairs. Then, we used enOsCas12f1 to edit the promoter and 5′ UTR of the *OsDREB1C* gene, demonstrating that enOsCas12f1 could stably produce base deletion, mutant rice plants. Additionally, we fused the transcriptional activation domain TV with the dead enOsCas12f1 to enhance the expression of the target gene *OsIPA1*. Our study demonstrates that enOsCas12f1 can be utilized for rice gene modification, thereby expanding the toolbox for rice gene editing.

## 1. Introduction

The Clustered Regularly Interspaced Short Palindromic Repeats (CRISPR)-associated protein (CRISPR/Cas) is derived from an adaptive immune system in bacteria [1,2]. The guide RNA (gRNA) and Cas nuclease form a complex and recognize the target sequence via base pairing between the gRNA and the target sequence. When a protospacer adjacent motif (PAM) is present in the target sites, the Cas nuclease cleaves double-stranded DNA (dsDNA), resulting in double-strand breaks (DSBs). These DSBs are subsequently repaired by homologous recombination (HDR) or non-homologous end joining repair (NHEJ) to achieve gene modification [3]. Currently, the most widely used genome editing tools are CRISPR/Cas9 and Cas12a, which identify NGG and T-rich PAMs, respectively [4,5]. Several studies have developed xCas9, Cas9-NG, and SpRY-Cas9 variants through protein engineering, expanding the targeting range of genome editing [6,7,8,9]. In addition, by fusing Cas Nickase (nCas) or dead Cas9 (dCas) variants with deaminases, reverse transcriptases, or transcriptional activators, a variety of editing tools have been developed, such as base editors, prime editors, and gene activations [10,11,12,13,14]. These techniques, developed for the targeted modification of the genome, have been used for improving crop yields, quality, stress tolerance, and disease resistance [15].

During the genome editing process, the vectors are delivered to the host cells. However, the large size of CRISPR/Cas9 or Cas12a systems (>1300 amino acids) makes delivery difficult, especially in the assembly of viral vectors with small carrying capacities for foreign genes [11,16,17]. Through deep sequencing and comparisons, a series of Cas14 (Cas12f1) proteins were discovered, which share structural similarities with Cas9 and Cas12 but are smaller in size [18,19,20]. Cas12f1 belongs to the class II type V-F Cas protein family, which consists of proteins ranging in size from 400 to 700 amino acids. It primarily utilizes the RuvC domain to cleave the target sequence and usually acts as a dimer [18]. A variety of Cas12f proteins exhibit genome editing abilities and can recognize diverse PAM sequences in prokaryotes and eukaryotes [20,21]. For instance, Cas12f1 from uncultured *Archaea* (Un1Cas12f1) contains 529 amino acids that recognize the TTTD PAM sequence. By modifying the structure and length of gRNA, editing efficiency can be improved, making it a successful tool for genome editing in plants and animals [22,23]. AsCas12f1, derived from *Acidbacillus sulfuroxidans*, is composed of 422 amino acids, recognizes T-rich PAM sequences, and has been proven to mediate efficient genome editing in animal cells [24,25]. The variants AsCas12f-YHAM and AsCas12f-HKRA significantly enhance the efficiency of genome editing in animal cells and have been successfully applied to rice genome editing [26,27]. SpCas12f1 from *Syntrophomonas palmitatica* is capable of genome editing in animal cells, and efficient genome editing in rice and maize can also be achieved through high-temperature treatment [25,28]. Cncas12f1 (*Clostridium novyi*) and RhCas12f1 (*Ruminiclostridium herbifermentans*) recognize C-rich PAMs and can perform genome editing in animal cells, and their editing efficiency can be improved by modifying the gRNA structure or protein sequence [29,30].

OsCas12f1 from *Oscillibacter* sp., consisting of 433 amino acids, shows high editing efficiency in mammalian cells. Its enhanced variant, enOsCas12f1, which was developed by protein engineering, significantly improved editing efficiency. Furthermore, the fusion of dead enOsCas12f1 (which has lost the ability to cleave double-stranded DNA) with an activator can increase gene expression in animal cells [30]. enOsCas12f1 has been used to generate exon skipping for Duchenne muscular dystrophy therapy in a humanized mouse model [31]. However, the gene editing efficiency of OsCas12f1 in rice is yet to be evaluated.

In this study, we constructed a CRISPR/enOsCas12f1 system with codons optimized for expression in rice and evaluated its editing efficiency in rice protoplasts. In addition, albino leaves were observed in enOsCas12f1-mediated *OsPDS* gene knockout rice plants, and the dead enOsCas12f1-based gene activator increases the expression level of the target gene in stably transformed rice plants. These results indicate that enOsCas12f1 can perform gene modification in rice, providing a new tool for basic research and molecular breeding.

## 2. Results

### 2.1. enOsCas12f1 Exhibits Cleavage Activity in Rice Protoplasts

**Figure 1:**
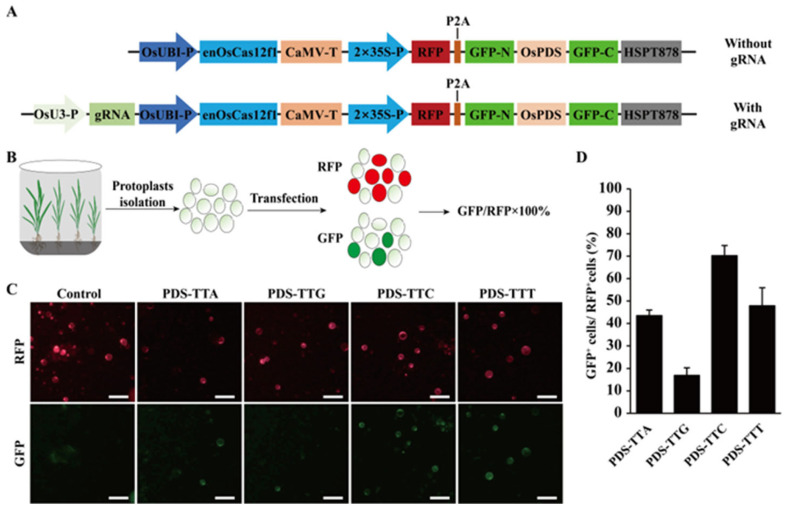
Detection of cleavage efficiency in rice protoplasts using a fluorescent reporting system. (**A**) The schematic of the SSA-based fluorescent reporter system construct used in this study. The construct without gRNA served as the control. CAMV-T, terminator from *CaMV*; HSPT878, *Arabidopsis thaliana* heat shock protein 18.2 gene terminator. (**B**) Scheme of enOsCas12f1 cleavage examined with SSA reporter. (**C**) Detection of protoplast fluorescence signal with fluorescence microscope; TTA, TTG, TTC, and TTT represent PAM sequences. Scale bar = 50 µm. (**D**) Statistical results of cleavage efficiency; values and error bars represent mean and s.d. (n = 3).

To evaluate the efficiency of enOsCas12f1 in cleaving dsDNA in rice, a fluorescence reporter system based on single-strand annealing (SSA) was constructed according to a previous report [32]. The GFP coding sequence was segmented into *GFP-N* and *GFP-C*, which share an overlapping region measuring 241bp. The *OsPDS* gene fragment was inserted between *GFP-N* and *GFP-C* fragments as a target sequence. The chimeric *GFP-N-OsPDS-GFP-C* and *RFP* sequences were linked by a P2A self-cleaving peptide and controlled by 2×*35S* promoter (Figure 1A and Figure S1). The rice codon-optimized *enOsCas12f1* was driven by the rice *OsUBI* promoter, and gRNA transcription was driven by the rice *OsU3* promoter (Figure 1A and Figure S2). Four gRNAs were designed to recognize TTN PAMs of the *OsPDS* gene. The vector was transferred into rice protoplasts using the PEG-mediated method. A vector without gRNA was used as the control (Figure 1A).

The red fluorescence signal indicated that the plasmid vector was transferred into rice protoplasts. If CRISPR/enOsCas12f1 targets the *OsPDS* fragment and generates double-strand breaks (DSBs), the presence of overlapping sequences between the two GFP segments would allow the formation of a complete GFP fluorescent protein through SSA (Figure 1B). After two days of incubation, the number of protoplasts expressing red fluorescence and green fluorescence was counted under a fluorescence microscope (Figure 1B).

A red fluorescence signal can only be obtained via the transformation of protoplasts by a gRNA-free vector. The four vectors containing gRNA showed red and green fluorescence after transforming the protoplasts (Figure 1C). The highest ratio was 70.19% when transforming the gRNA that recognizes the TTC PAM, and the lowest was 16.87% when recognizing the TTG PAM (Figure 1D). This indicates that enOsCas12f1 can cleave dsDNA in rice protoplasts.

### 2.2. enOsCas12f1 Enables Gene Editing in Stably Transformed Rice Plants

**Figure 2:**
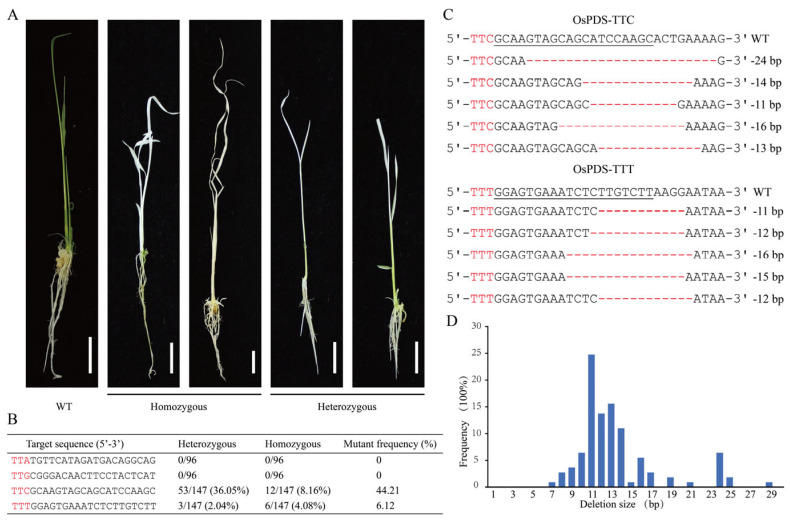
Detection of gene editing efficiency in stably transformed rice. (**A**) Albino leaves observed in OsPDS-TTC/TTT plants; from left to right are the wild-type plants, as well as homozygous and heterozygous mutants. Scale bar = 1 cm. (**B**) Summary of editing efficiencies of the transgenic rice plants; target sequences in *OsPDS* are shown, with PAM sequences highlighted in red. (**C**) Sequence alignment of wild-type and mutant plants; PAM sequences are highlighted in red, the target sequence is underlined, the red line represents base deletions, and the number of base deletions is shown on the right. (**D**) Frequencies of each deletion size in mutant plants.

To explore the endogenous genome-editing capability of enOsCas12f1 in stably transformed rice plants, the above gRNAs, targeting the *OsPDS* gene, were assembled into two vectors recognizing TTA/TTG and TTC/TTT PAMs, respectively. Then, stably transformed rice plants were obtained via *Agrobacterium*-mediated transformation. Albino or striped rice plants were also observed when treated with vectors that recognized TTC/TTT PAMs (Figure 2A). Then, DNA was extracted from transgenic plants and subjected to Hi-TOM sequence analysis. The results showed that the highest editing efficiency was achieved at the TTC PAM target sequence (44.21%), while the editing efficiency at the TTT PAM was 6.12% (Figure 2B). This indicates that enOsCas12f1 can perform gene editing in stably transformed rice plants, and TTC is the optimal PAM sequence. In addition, sequence analysis showed that all mutations generated by enOsCas12f1 were base deletions ranging in size from 7 to 29 bp (Figure 2C,D).

### 2.3. Application of enOsCas12f1 in Editing the Promoter Region of Rice

**Figure 3:**
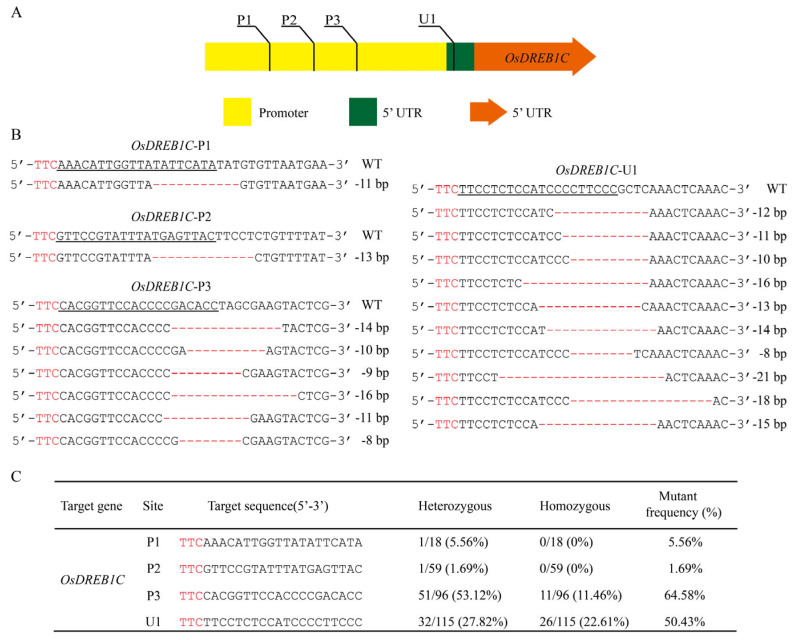
Editing of the gene regulatory regions of *OsDREB1C* was mediated by enOsCas12f1. (**A**) The relative position of the target sites in the gene promoter. Target sequences P1, P2, and P3 are located at the promoter, and U1 is located at the 5′ UTR. (**B**) Sequence alignment of wild-type and mutant plants; PAM sequences are highlighted in red, the target sequence is underlined, the red line represents base deletions, and the number of bases deleted is on the right. (**C**) Summary of editing efficiencies of the transgenic rice plants; PAM sequences are highlighted in red.

Since enOsCas12f1 has the potential to generate DNA deletions, it can be applied to the study of promoter regulatory sequences [33]. Four gRNAs were designed to target the promoter and 5′ untranslated region (5′-UTR) of *OsDREB1C* (*LOC_Os06g03670*), generating transgenic rice plants (Figure 3A). The sequence analysis results showed that deletions occurred in all target sites (Figure 3B). High editing efficiencies were observed at P3 and U1 target sequences, reaching 64.58% and 50.43%, respectively (Figure 3C). These sequence deletion mutants can be used to examine the function of regulatory regions in future studies.

### 2.4. IPA1 Activated by Fusion of Dead enOsCas12f1 with Transcriptional Activation Domain

**Figure 4:**
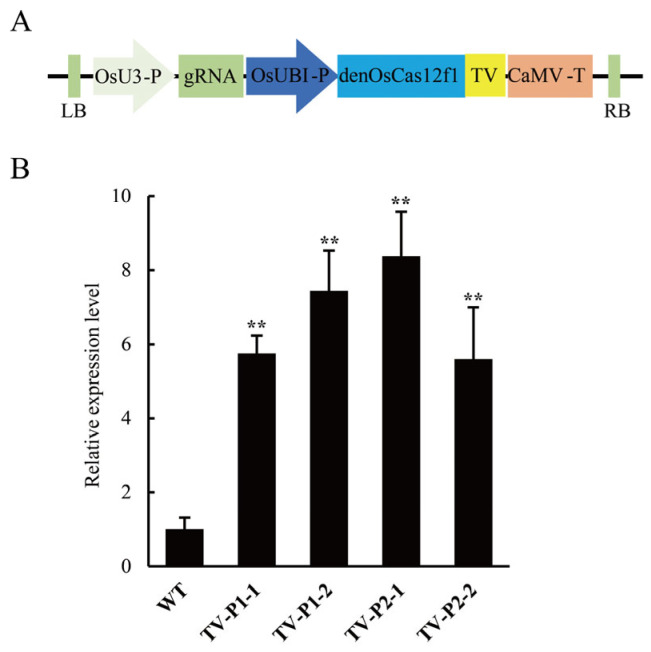
The *OsIPA1* gene was activated by the denOsCas12f1-TV system. (**A**) Schematic of the denOsCas12f1-TV construct used in this study. LB, left border of the T-DNA insertion; RB, right border of the T-DNA insertion. (**B**) *OsIPA1* expression increased due to denOsCas12f1-TV, TV-P1-1, and TV-P1-2; independent rice plants were obtained via the genetic transformation of the TV-P1, TV-P2-1, and TV-P2-2 vectors; and independent rice plants were obtained by the genetic transformation of the TV-P2 vector. The error bar represents s.d. (n = 3); ** *p* < 0.01 (Student’s *t*-test). *OsAct1* was used as a control to normalize all data.

We also generated dead enOsCas12f1 (denOsCas12f1, D228A/D406A) and fused it with the trans-activator domain TV (denOsCas12f1-6TAL-VP128) (Figure 4A and Figure S3). Two gRNAs were designed to target the promoter of *OsIPA1* (TV-P1 and TV-P2), producing stable rice plants (Figure S4). The qRT-PCR results showed that the expression of the *OsIPA1* (*LOC_Os08g39890*) gene increased 5.60–8.38 fold in transgenic rice plants compared to WT rice plants (Figure 4B).

## 3. Discussion

The CRISPR/Cas gene editing tools have been widely used in the genetic modification of animals, plants, and microorganisms. Cas12f1 is a recently discovered gene-editing enzyme, which shows great potential due to its small size and variable PAM recognition range [20,21,25,34]. OsCas12f1 from *Oscillibacter* sp. recognizes the TTN PAM. Its modified variant, enOsCas12f1, demonstrates high editing efficiency in animal cells [30,31], but its applicability in plant gene editing remains to be verified.

In this study, we first optimized the enOsCas12f1 codons and verified its cleavage efficiency in rice protoplasts using the SSA-based fluorescent reporter system. The results showed that it had cleavage activity at the TTN PAMs sites in rice protoplasts. The highest cutting efficiency is shown in TTC PAMs, like previous studies in animal cells, where enOsCas12f1’s preference for PAM sequences is 5′-TTC > 5′-TTA > 5′-TTT > 5′-TTG [30]. However, in stably transformed rice plants, only TTC and TTT demonstrated gene editing capabilities, which limited their applications in gene editing. According to previous research, the optimum working temperature for enOsCas12f1 ranges from 37 to 42 °C [30]. Similarly, SpCas12f1, which also requires a higher working temperature and undergoes heat treatment at 45 °C, can significantly improve the gene editing efficiency in maize [25]. Furthermore, studies have found that SpCas12f1 works well in rice by maintaining the transformation process at a temperature above 30 °C and keeping the light constant during callus proliferation [28]. In this study, the genetic transformation of rice was performed at 32 °C, and the effect of higher temperature treatments on the efficiency of enOsCas12f1 remains to be studied in future research.

The cleavage site of Cas12f1 is often distal to the PAM, as it is located outside the target sequence. Studies have suggested that the PAM sequence and target sequence remain intact during initial gene editing. This allows the Cas12f1 protein to continuously recognize and cleave the target sequence, resulting in deletion mutations rather than insertions or substitutions [22,23,26]. This finding is consistent with our study; all mutations produced by enOsCas12f1 were deletions ranging in size from 7 to 29 bp. This suggests that enOsCas12f1 is a suitable gene editing tool for generating multi-base deletions. We also observed various fragment deletion mutants during the editing of rice gene regulatory regions. This indicates that enOsCas12f1 has potential applications in studying the functional regions of promoters.

The fusion of the dead Cas protein and the transcriptional activation domain provides a new way of controlling the activity of target genes of interest in the absence of DSB [35]. In this study, the fusion of the denOsCas12f1 protein with TV increased the expression level of the target gene in rice, showing the potential of enOsCas12f1 for gene activation.

In summary, our study demonstrates that enOsCas12f1 is a valuable tool for rice genome modification and is especially suitable for creating fragment deletion mutants. Further optimization is needed to expand the recognition ranges of PAMs and improve editing efficiency. Additionally, since Cas12f1 generates DSBs in the form of dimers, it can only produce inactive versions of the protein rather than nicked versions of the enzyme [20]. This limitation poses a challenge to the integration of tools such as base substitution and prime editing, necessitating further research.

## 4. Materials and Methods

### 4.1. Construction of CRISPR/enOsCas12f1-Related Vectors

The backbone vector was derived from pCAMV1300-UBI-Cas9. The codon-optimized enOsCas12f1 sequence was positioned between the *OsUBI* promoter and the CAMV terminator. The *GFP* coding sequence was split by PCR into two overlapping segments measuring 240 bp, which were fused to the *RFP* by the peptide P2A. gRNA construction was carried out following the method described in a previous study [36]. The denOsCas12f1 fused with TV (a combination of VP128 with six copies of the TALE TAD motif) [37] was generated via overlap PCR (Table S1). The vectors were produced by seamless cloning.

### 4.2. Isolation and Transformation of Rice Protoplasts

The dehulled seeds of the rice variety used in this study—Xinfeng 2 (*Oryza sativa* L. ssp. *japonica*)—were sterilized with 70% ethanol for 1 min and then placed in 30 mL of 2.5% sodium hypochlorite for 30 min while being shaken, after which the seeds were washed 5 times using sterile water. After sterilization, seeds were placed on a 1/2 MS medium and cultured in darkness at 28 °C for 15 days. Protoplasts were then isolated using leaf sheaths, following the method described in previous studies [38]. The constructed vectors were extracted from DH5α *E. coli* cells using a plasmid extraction kit, and 5 μg of DNA was used for each transformation. After protoplast transformation, which was performed using the PEG method, protoplasts were incubated in the dark at 28 °C for 36–48 h and then observed under a fluorescence microscope. The red and green fluorescence signals were counted to calculate the cleavage efficiency. Each transformation was repeated 3 times.

### 4.3. Genetic Transformation of Rice

The genetic transformation of rice was achieved by following established protocols with slight modifications [39]. After 15 days of callus induction, the rice was infected with *Agrobacterium* and co-cultured for 3 days before being subjected to a selection culture containing 50 µg/mL hygromycin. After 30 days of selection, the cultures were transferred to a differentiation medium. Once seedlings emerged, they were moved to a rooting medium. All genetic transformation steps were performed at 32 °C.

### 4.4. Genotyping of Transgenic Plants

After extracting DNA from rice leaves, sequencing was performed using Hi-TOM, following the method described in previous studies [40]. A 100 bp fragment flanking the target sequence was amplified by PCR (Table S1), and barcode and index sequences were added through two rounds of PCR. The PCR products were recovered using a gel extraction kit and subsequently subjected to NGS sequencing. The sequencing results were analyzed using the Hi-TOM platform.

### 4.5. RNA Extraction and qRT-PCR for Evaluating Transcriptional Activation

Total RNA was extracted from rice plants using RNAiso Plus (Takara). Subsequently, 1 µg of RNA was used for cDNA synthesis using the 1st Strand cDNA Synthesis Kit (Takara). qPCR was performed according to the qPCR SYBR Green Master Mix protocol (Yeasen). The results were normalized with *OsAct1*, and fold changes were calculated using the 2^−ΔΔCt^ method.

## Data Availability

The original contributions presented in this study are included in the article. Further inquiries can be directed to the corresponding author.

## Supplementary Materials

The following supporting information can be downloaded at https://www.mdpi.com/article/10.3390/plants14142100/s1, Figure S1: the sequence of SSA-based fluorescence reporter system; Figure S2: the sequence of rice codon-optimized *enOsCas12f1* and *enOs-gRNA*; Figure S3: the sequence of *enOsCas12f1-TV*; Figure S4: schematic of the *OsIPA1* gene activation target sites; Table S1: primer sequences used in this study.
