# Supplementary material for: Miniature enOsCas12f1 Enables Targeted Genome Editing in Rice
Source: Plants (Basel). 2025 Jul 8;14(14):2100. doi: 10.3390/plants14142100 (PMC12299486; doi:10.3390/plants14142100)
Supplement: Supplementary file 1 [file plants-14-02100-s001.zip › Supplemental Data.pdf]

Supplemental Materials:

>2×35S-COR47-RFP-P2A-GFP-N-OsPDS-GFP-C-HSPT878

CCTACTCCAAAAATGTCAAAGATACAGTCTCAGAAGACCAAAGGGCTATTGAGACTT
TTCAACAAAGGGTAATTTCGGGAAACCTCCTCGGATTCCATTGCCCAGCTATCTGTC
ACTTCATCGAAAGGACAGTAGAAAAGGAAGGTGGCTCCTACAAATGCCATCATTGCG
ATAAAGGAAAGGCTATCATTCAAGATGCCTCTGCCGACAGTGGTCCCAAAGATGGAC
CCCCACCCACGAGGAGCATCGTGGAAAAAGAAGACGTTCCAACCACGTCTTCAAAGC
AAGTGGATTGATGTGACATCTCCACTGACGTAAGGGATGACGCACAATCCCACCCCT
ACTCCAAAAATGTCAAAGATACAGTCTCAGAAGACCAAAGGGCTATTGAGACTTTTC
AACAAAGGGTAATTTCGGGAAACCTCCTCGGATTCCATTGCCCAGCTATCTGTCACT
TCATCGAAAGGACAGTAGAAAAGGAAGGTGGCTCCTACAAATGCCATCATTGCGATA
AAGGAAAGGCTATCATTCAAGATGCCTCTGCCGACAGTGGTCCCAAAGATGGACCCC
CACCCACGAGGAGCATCGTGGAAAAAGAAGACGTTCCAACCACGTCTTCAAAGCAAG
TGGATTGATGTGACATCTCCACTGACGTAAGGGATGACGCACAATCCCACTATCCTT
CGCAAGACCCTTCCTCTATATAAGGAAGTTCATTTCATTTGGAGAGGACAGCCCACA
AACATTACTCATTCACAAAACCATCTTAAAGCAACTACACAAGTCTTGAAATTTTCT
CATATTTTCTATTTACTATATAAACTTTTAATCAAATCAAGATTAAAGATGGCCTCC
TCCGAGGACGTCATCAAGGAGTTCATGCGCTTCAAGGTGCGCATGGAGGGCTCCGTG
AACGGCCACGAGTTCGAGATCGAGGGCGAGGGCGAGGGCCGCCCCTACGAGGGCACC
CAGACCGCCAAGCTGAAGGTGACCAAGGGCGGCCCCCTGCCCTTCGCCTGGGACATC
CTGTCCCCTCAGTTCCAGTACGGCTCCAAGGCCTACGTGAAGCACCCCGCCGACATC
CCCGACTACTTGAAGCTGTCCTTCCCCGAGGGCTTCAAGTGGGAGCGCGTGATGAAC
TTCGAGGACGGCGGCGTGGTGACCGTGACCCAGGACTCCTCCCTGCAGGACGGCGAG
TTCATCTACAAGGTGAAGCTGCGCGGCACCAACTTCCCCTCCGACGGCCCCGTAATG
CAGAAGAAGACGATGGGCTGGGAGGCCTCCACCGAGCGGATGTACCCCGAGGACGGC
GCCCTGAAGGGCGAGATCAAGATGAGGCTGAAGCTGAAGGACGGCGGCCACTACGAC
GCCGAGGTCAAGACCACCTACATGGCCAAGAAGCCCGTGCAGCTGCCCGGCGCCTAC
AAGACCGACATCAAGCTGGACATCACCTCCCACAACGAGGACTACACCATCGTGGAA
CAGTACGAGCGCGCCGAGGGCCGCCACTCCACCGGCGCCGGAAGCGGAGCTACTAAC
TTCAGCCTGCTGAAGCAGGCTGGTGACGTGGAGGAGAACCCTGGACCTATGGTGAGC
AAGGGCGAGGAGCTGTTCACCGGGGTGGTGCCCATCCTGGTCGAGCTGGACGGCGAC
GTAAACGGCCACAAGTTCAGCGTGTCCGGCGAGGGCGAGGGCGATGCCACCTACGGC
AAGCTGACCCTGAAGTTCATCTGCACCACCGGCAAGCTGCCCGTGCCCTGGCCCACC
CTCGTGACCACCTTCACCTACGGCGTGCAGTGCTTCAGCCGCTACCCCGACCACATG
AAGCAGCACGACTTCTTCAAGTCCGCCATGCCCGAAGGCTACGTCCAGGAGCGCACC
ATCTTCTTCAAGGACGACGGCAACTACAAGACCCGCGCCGAGGTGAAGTTCGAGGGC
GACACCCTGGTGAACCGCATCGAGCTGAAGGGCATCGACTTCAAGGAGGACGGCAAC
ATCCTGGGGCACAAGCTGGAGTACAACTACAACAGCCACAACGTCTATATCATGGCC
GACAAGCAGAAGTGATAAGGGGATACTGGCTGCCTGTCATCTATGAACATAACTGGA
ACCAGCCAAGCAAGATCTTTTGCGGGACAACTTCCTACTCATAGGTGCTTCGCAAGT
AGCAGCATCCAAGCACTGAAAAGTAGTCAGCATGTGAGCTTTGGAGTGAAATCTCTT
GTCTTAAGGAATAAAGGAAAAAGATTCCGTCGGAGGCTCGGTGCTCTACAGGTTCAA
CCTTTGTACTCTATTATTGCCTCACATTCCATCTCTTGTGAAAATATATTTGATTGG

CTTTTCTGCAGGTTGTTTGCCAGGACTTTCCAAGACCTCCACTAGAAAACACAATAA
ACTTTTTGGAAGCTGGACAACTATCTTCAATCCTCGAGAGAAGCAGCACGACTTCTT
CAAGTCCGCCATGCCCGAAGGCTACGTCCAGGAGCGCACCATCTTCTTCAAGGACGA
CGGCAACTACAAGACCCGCGCCGAGGTGAAGTTCGAGGGCGACACCCTGGTGAACCG
CATCGAGCTGAAGGGCATCGACTTCAAGGAGGACGGCAACATCCTGGGGCACAAGCT
GGAGTACAACTACAACAGCCACAACGTCTATATCATGGCCGACAAGCAGAAGAACGG
CATCAAGGTGAACTTCAAGATCCGCCACAACATCGAGGACGGCAGCGTGCAGCTCGC
CGACCACTACCAGCAGAACACCCCCATCGGCGACGGCCCCGTGCTGCTGCCCGACAA
CCACTACCTGAGCACCCAGTCCGCCCTGAGCAAAGACCCCAACGAGAAGCGCGATCA
CATGGTCCTGCTGGAGTTCGTGACCGCCGCCGGGATCACTCACGGCATGGACGAGCT
GTACAAGTAAATATGAAGATGAAGATGAAATATTTGGTGTGTCAAATAAAAAGCTTG
TGTGCTTAAGTTTGTGTTTTTTTCTTGGCTTGTTGTGTTATGAATTTGTGGCTTTTT
CTAATATTAAATGAATGTAAGATCTCATTATAATGAATAAACAAATGTTTCTATAAT
CCATTGTGAATGTTTTGTTGGATCTCTTCTGCAGCATATAACTACTGTATGTGCTAT
GGTATGGACTATGGAATATGATTAAAGATAAGATGGGCTCATAGAGTAAAACGAGGC
GAGGGACCTATAAACCTCCCTTCATCATGCTATTTCATGATCTATTTTATAAAATAA
AGATGTAGAAAAAAGTAAGCGTAATAACCGCAAAACAAATGATTTAAAACATGGCAC
ATAATGAGGAGATTAAGTTCGGTTTACGTTTATTTTAGTACTAATTGTAACGTGAGA
CTACGTATCGGGAATCGCCTAATTAAAGCATTAATGCGAACCTGATTAGATTCACCG
ACCCTCCTATCGTGACGACCTTTCTGTTTCTTAGAATTTTTTGGTAGTCTATGTACT
AATAATGTCAGCTTCGTATTTATTTCATAAGCAATTTGCATTTGCAATTTGTTTTTT
ACTTTTATTTTTATTGTATTGTGGAATGTGGACTCGTACCAACATGAAGTTATATAC
CACCAAAAAAATTACAGTTAGTCAAAAGATTCACGAGTGAGAGCTACTTATGATTGT
CTTTTACGTATATGTCTAATTGTCTATTTGCTCAATAATCTTTGTACTTTCTTTTGT
CGTTGATAAAATCACAAAGTTCCAAAAGTAATCGAATGATTTGCTTTTAAGAAAAGA
AGAGCTCAATAATTCAACATATATCTGTACACA

Figure S1. The sequence of SSA based fluorescence reporter system

2×*35S*: enhanced *35S* promoter from *CaMV*, COR47: enhancor from *Arabidopsis thaliana* cold-regulated 47 gene 5'-untranslated region, RFP: red fluorescent protein coding sequence, P2A: self-cleaving peptide, GFP-N/ GFP-C: red fluorescent protein coding sequence divided 2 fragment, the yellow background letters represent an overlapping 241 bp sequence, HSPT878: *Arabidopsis thaliana* heat shock protein 18.2 gene terminator.

>NLS-enOsCas12f1-NLS
ATGGCCCCTAAGAAGAAGAGAAAGGTCGGTATTCACGGCGTTCCTGCGGCGATGGGC
AAGGGAGTCTTGGCAAAGGTGATGAAGTATGAATTGAGATATTTGGATGGTTGCGGG
GATTTTAGCAACATGCAAGAACAAGTCTGGGCCCTTCAACGGCAGACGCGGGAGATC
CTTAATCGCAGTATCCAGATTGCCTTTCAATGGCGCTGCGCGAATAGCGAACATCAC
CGGAAGACCGGAGAGTATCTTGATCTGAAGACCGAGACAGGTTATAAGCGCCTGGAT
GGCCATATCTACAACTGCCTGAAGGGCCAGTACGAGGATATGGCAACGTCCAACCTG
AACGCCACAATTCAAAAGGCCTGGAAGAAGTACAACAGCTCCAAGAAGGAGATTCTC
CGGGGCTCAATGTCCATCCCGTCTTATAAGATGAATCAGCCTCTTCGCCTCGACAAG
AACACCGTGAAGCTGTCCGAGGGTGAGAGGAACCCGATTGTGACACTGACACTCTTC

TCTGACAAGTTTAAGAGAGCACAGGGTGTCTCGAATGTGAAGTTCTCTATGCCTCTG
CATGATGGAACGCAGCGCGCGATCTTCGCGAACTTGATGAACGGAACCTATCAGCTG
GGTGAGTGCCAACTTGTGTATAAGCGCCCTAAGTGGTTCCTCTTCGTGACATACAAG
TTCCCTCCAGTGGAACACCCTCTCGACCCAGACAAGATCCTGGGAGTCGATATGGGT
GAGGCTTGCGCGCTCTATGCGTCTACATTTGGAGAGCACGGTTACCTGAAGATCGAC
GGAGGTGAAATCACAAAGTACGCTAAGAAGATGGAAGCGCGCATCAGGTCGATGCAA
AAGCAAGCTGCTCATTGCGGAGAAGGTAGGATCGGACATGGCACGAAGACCAGGGTG
TCTGTGGTTTACCAGGCCAAGGACAAGGTGGCGCGGTTTAGAGACACCATCAACCAT
CGGTACTCCAAGGCGCTCATCGACTATGCGCTGAAGAATCAGTGCGGAACGATTCAG
ATGGAGGACCTCACCGGTATCAAGGAGGACACCGGCTTTCCAAAGTTCCTGAGGCAC
TGGACGTACTACGATCTCCAATCCAAGATCGAGGCAAAGGCCGCAGAGCACGGGATT
CAAGTGGTTAAGATCAATCCCCGGCACACCAGTCAGAGATGCTCGCGGTGCGGTCAC
ATCGATAAGGCCAATAGGACGAGCCAAGCAGACTTCTGCTGCACCAAGTGCGGTTTT
TCGGCGAACGCCGACTTTAACGCCTCTCAGAACATCTCGATCCGGAATATCGACAAG
ATTATCGCTAAGGCAATCGGAGCCAACAGAAAGCAAACAAAGAGACCAGCGGCAACC
AAGAAGGCAGGACAAGCGAAGAAGAAGAAGTAG

>OsU3-enOsgRNA-Target

AAGGAATCTTTAAACATACGAACAGATCACTTAAAGTTCTTCTGAAGCAACTTAAAG
TTATCAGGCATGCATGGATCTTGGAGGAATCAGATGTGCAGTCAGGGACCATAGCAC
AAGACAGGCGTCTTCTACTGGTGCTACCAGCAAATGCTGGAAGCCGGGAACACTGGG
TACGTCGGAAACCACGTGATGTGAAGAAGTAAGATAAACTGTAGGAGAAAAGCATTT
CGTAGTGGGCCATGAAGCCTTTCAGGACATGTATTGCAGTATGGGCCGGCCCATTAC
GCAATTGGACGACAACAAAGACTAGTATTAGTACCACCTCGGCTATCCACATAGATC
AAAGCTGATTTAAAAGAGTTGTGCAGATGATCCGTGGCAAGGGCCGACTTCCCGGCC
CAAAATCGAGACAGTAGCCGTAAAACGTTGAGTTTCAGCGTGGGCGACACACTCGAA
AAGGTTAAGATATGCACATAGTAATCCGTGCATGAGCCGCGAAAGCGGCTTGAAGGN
NNNNNNNNNNNNNNNNNNN

Figure S2. The sequence of enOsCas12f1 and enOs-gRNA

NLS: Nuclear Localization Signal, OsU3: rice *OsU3* promoter, Target: 20 bp target sequence follow gRNA

> NLS-denOsCas12f1(D228A/D406A)-NLS-TV

ATGGCCCCTAAGAAGAAGAGAAAGGTCGGTATTCACGGCGTTCCTGCGGCCATGGGC
AAGGGAGTCTTGGCAAAGGTGATGAAGTATGAATTGAGATATTTGGATGGTTGCGGG
GATTTTAGCAACATGCAAGAACAAGTCTGGGCCCTTCAACGGCAGACGCGGGAGATC
CTTAATCGCAGTATCCAGATTGCCTTTCAATGGCGCTGCGCGAATAGCGAACATCAC
CGGAAGACCGGAGAGTATCTTGATCTGAAGACCGAGACAGGTTATAAGCGCCTGGAT
GGCCATATCTACAACTGCCTGAAGGGCCAGTACGAGGATATGGCAACGTCCAACCTG
AACGCCACAATTCAAAAGGCCTGGAAGAAGTACAACAGCTCCAAGAAGGAGATTCTC
CGGGGCTCAATGTCCATCCCGTCTTATAAGATGAATCAGCCTCTTCGCCTCGACAAG
AACACCGTGAAGCTGTCCGAGGGTGAGAGGAACCCGATTGTGACACTGACACTCTTC
TCTGACAAGTTTAAGAGAGCACAGGGTGTCTCGAATGTGAAGTTCTCTATGCCTCTG
CATGATGGAACGCAGCGCGCGATCTTCGCGAACTTGATGAACGGAACCTATCAGCTG

GGTGAGTGCCAACTTGTGTATAAGCGCCCTAAGTGGTTCCTCTTCGTGACATACAAG
TTCCCTCCAGTGGAACACCCTCTCGACCCAGACAAGATCCTGGGAGTCGCGATGGGT
GAGGCTTGCGCGCTCTATGCGTCTACATTTGGAGAGCACGGTTACCTGAAGATCGAC
GGAGGTGAAATCACAAAGTACGCTAAGAAGATGGAAGCGCGCATCAGGTCGATGCAA
AAGCAAGCTGCTCATTGCGGAGAAGGTAGGATCGGACATGGCACGAAGACCAGGGTG
TCTGTGGTTTACCAGGCCAAGGACAAGGTGGCGCGGTTTAGAGACACCATCAACCAT
CGGTACTCCAAGGCGCTCATCGACTATGCGCTGAAGAATCAGTGCGGAACGATTCAG
ATGGAGGACCTCACCGGTATCAAGGAGGACACCGGCTTTCCAAAGTTCCTGAGGCAC
TGGACGTACTACGATCTCCAATCCAAGATCGAGGCAAAGGCCGCAGAGCACGGATT
CAAGTGGTTAAGATCAATCCCCGGCACACCAGTCAGAGATGCTCGCGGTGCGGTCAC
ATCGATAAGGCCAATAGGACGAGCCAAGCAGACTTCTGCTGCACCAAGTGCGGTTTT
TCGGCGAACGCCGCCTTTAACGCCTCTCAGAACATCTCGATCCGGAATATCGACAAG
ATTATCGCTAAGGCAATCGGAGCCAACAGAAAGCAAACAAAGAGACCAGCGGCAACC
AAGAAGGCAGGACAAGCGAAGAAGAAGAAGTCAGGCTCAGTCGACTTGCTTGATCCG
GGGACACCAATGGACGCGGACCTGGTGGCTTCATCGACCGTGGTTTGGGAACAGGAC
GCCGATCCATTCGCCGGGACCGCCGATGACTTTCCTGCTTTTAATGAGGAAGAGTTG
GCTTGGCTGATGGAACTCCTGCCGCAGGGCGGCTCAGGGGGTCTCCTTGACCCCGGC
ACCCCCATGGACGCTGACCTCGTTGCAAGTTCGACGGTTGTTTGGGAGCAAGATGCA
GATCCGTTTGCGGGTACAGCTGATGACTTTCCAGCCTTCAACGAAGAGGAGCTGGCA
TGGCTTATGGAGCTTCTGCCTCAGGCCAGAGGAGGGTCGGGTGGGCTCTTGGATCCT
GGGACGCCGATGGATGCCGACCTTGTCGCGTCCTCTACTGTGGTTTGGGAGCAGGAC
GCCGACCCGTTCGCTGGTACTGCTGATGACTTTCCGGCCTTTAATGAGGAGGAACTC
GCTTGGCTCATGGAGTTGTTGCCACAAGGGGGTAGTGGCGGTCTTCTGGACCCGGGA
ACGCCTATGGATGCTGACTTGGTGGCATCGAGCACCGTCGTCTGGGAACAGGATGCG
GACCCGTTTGCTGGTACCGCTGATGATTTTCCGGCATTTAACGAGGAAGAATTGGCG
TGGCTCATGGAATTGCTTCCCCAGGCGAGAGGGGGTTCCGGTGGCTTGCTGGATCCG
GGAACGCCTATGGATGCCGATCTCGTGGCGTCGTCAACCGTGGTCTGGGAGCAAGAC
GCTGACCCGTTTGCCGGCACAGCAGATGATTTTCCAGCTTTCAATGAGGAAGAACTG
GCATGGTTGATGGAATTGCTTCCACAGGGAGGGAGCGGAGGCCTCCTCGATCCTGGA
ACACCTATGGACGCAGACCTGGTTGCCTCTTCAACTGTCGTGTGGGAACAAGATGCA
GACCCGTTCGCAGGTACAGCTGATGACTTCCCTGCCTTTAATGAGGAGGAACTCGCG
TGGCTCATGGAACTTTTGCCCCAAGCACGCGGTGGCTCTGGGGGTGGCGGATCGGGA
GGAGATGCGCTGGACGATTTCGACTTGGACATGTTGGGCTCAGACGCTTTGGACGAC
TTTGATCTCGACATGCTTGGGTCCGACGCATTGGATGATTTCGACCTTGACATGCTT
GGTTCCGACGCACTCGATGACTTCGATCTTGATATGCTCGCCCGCGGATCTGACGCT
CTCGACGACTTCGATCTTGACATGTTGGGCTCAGATGCGCTCGACGACTTTGATCTC
GATATGTTGGGTAGCGACGCACTCGATGACTTTGACTTGGATATGCTGGGGAGCGAC
GCCTTGGACGATTTCGATCTGGACATGCTGTAA

Figure S3. The sequence of enOsCas12f1-TV

NLS: Nuclear Localization Signal, GCG/GCC: D228A/D406A mutant sites, TV: the trans-activator domain

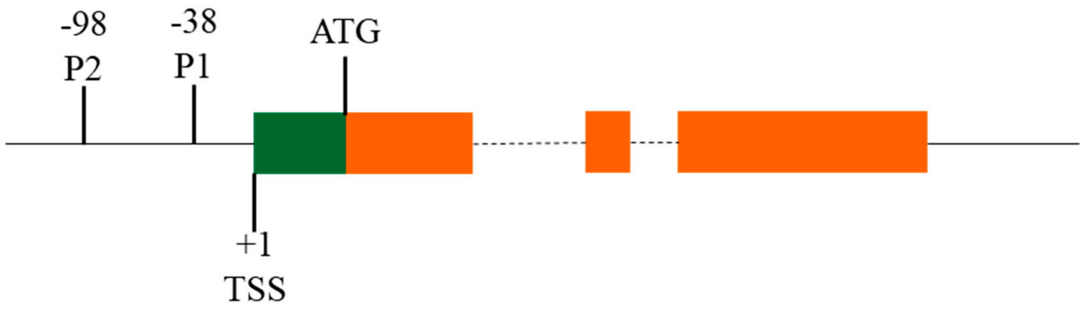

Figure S4. Schematic of the *OsIPA1* gene activation target sites

TSS: transcriptional start site, ATG: start codon, -38 and -98: The distance from the target site P1 and P2 to the transcription initiation site, The green rectangle represents the 5 ' -UTR, orange rectangle represents exons, ---- represents introns.

Table S1 Primer sequences used in this study.

Primer	Primer sequence (5'-3')	
PDS-Os-AF	AAGGTGTTCATAGATGACAGGCAG	PDS-TTA
PDS-Os-AR	AAAACTGCCTGTCATCTATGAACA	
PDS-Os-CF	AAGGGCAAGTAGCAGCATCCAAGC	PDS-TTC
PDS-Os-CR	AAAAGCTTGGATGCTGCTACTTGC	
PDS-Os-GF	AAGGCGGGACAACTTCCTACTCAT	PDS-TTG
PDS-Os-GR	AAAAATGAGTAGGAAGTTGTCCCG	
PDS-Os-TF	AAGGGGAGTGAAATCTCTTGTCTT	PDS-TTT
PDS-Os-TR	AAAAAAGACAAGAGATTTCACTCC	
GFP-PDS-F	GACAAGCAGAAGTGATAAGGGGATACTGGCTGCCTGTCATC	PCR for *OsPDS*
GFP-PDS-R	CGTGCTGCTTCTCTCGAGGATTGAAGATAGTTGTCCAGCTTCC	
H-PDS-F	GGAGTGAGTACGGTGTGCAGGAGTTGCTTCAGCATGGAT	Hi-TOM sequencing
H-PDS-R	GAGTTGGATGCTGGATGGAAGCTCACATGCTGACTAC	
H-PDS-F1	GGAGTGAGTACGGTGTGCCTGGAACCAGCCAAGCAAG	
H-PDS-R1	GAGTTGGATGCTGGATGGTAGAGCACCGAGCCTCCGAC	
DR-P1-F	AAGGAAACATTGGTTATATTCATAT	DREB1C-P1
DR-P1-R	AAAAATATGAATATAACCAATGTTT	
DR-P2-F	AAGGGTTCCGTATTTATGAGTTACT	DREB1C-P2
DR-P2-R	AAAAAGTAACTCATAAATACGGAAC	
DR-P3-F	AAGGCACGGTTCCACCCCGACACCT	DREB1C-P3
DR-P3-R	AAAAAGGTGTCGGGGTGGAACCGTG	
DR-U1-F	AAGGTTCCTCTCCATCCCCTTCCCG	DREB1C-U1
DR-U1-R	AAAACGGGAAGGGGATGGAGAGGAA	
H-DR-F1	GGAGTGAGTACGGTGTGCGATTTCACCCGTTGCAACGCACG	HI-TOM sequencing
H-DR-R1	GAGTTGGATGCTGGATGGGTCCATATTCATATAGATG	
H-DR-F2	GGAGTGAGTACGGTGTGCGGACAATACTAGAAAGACTTAC	
H-DR-R2	GAGTTGGATGCTGGATGGACCACTTCTGTGTGTGTATG	
H-DR-F3	GGAGTGAGTACGGTGTGCCGCTGTCACCTCACCCTCT	
H-DR-R3	GAGTTGGATGCTGGATGGACGGATGGAACGGTGGGATTC	
H-DR-F4	GGAGTGAGTACGGTGTGCCAAAATTCACACACCAAATCC	
H-DR-R4	GAGTTGGATGCTGGATGGGCTCGTAGTACTCCATGTCA	
IPA1-P1-F	AAGGTTATACCACAGCTGCAACAT	IPA1-P1
IPA1-P1-R	AAAAATGTTGCAGCTGTGGTATAA	
IPA1-P2-F	AAGGTACTGTGGGTGCAGTGTCAT	IPA1-P2
IPA1-P2-R	AAAAATGACACTGCACCCACAGTA	
IPA1-QF	TGCATTCCAAGGCTCCCCGC	qRT-PCR
IPA1-QR	TGCGGCAGCTGCGTTTTCCT	
OsActin-QF	CTTCATAGGAATGGAAGCTGCGGGTA	
OsActin-QR	CGACCACCTTGATCTTCATGCTGCTA	
dOs-F1	GGGAGTCGCGATGGGTGAGGCTTG	D228A/ D406A
dOs-R1	TCACCCATCGCGACTCCCAGGATC	
dOs-F2	CGAACGCCGCCTTTAACGCCTCTCA	

dOs-R2	GCGTTAAAGGCGGCGTTCGCCGAAA	
TV-F	ACAAGCGAAGAAGAAGAAGTCAGGCTCAGTCGACTTGCTTGATCC	TV
TV-R	CTGGTGATTTCAGCGTACCGAATTTACAGCATGTCCAGATCGAAA	
